# Warming alters non-trophic interactions in soft bottom habitats

**DOI:** 10.1007/s00442-025-05662-y

**Published:** 2025-02-06

**Authors:** Simona Laukaityte, Melanie J. Bishop, Laura L. Govers, Britas D. H. Klemens Eriksson

**Affiliations:** 1 Groningen Institute for Evolutionary Life-Sciences, GELIFES, University of Groningen, Nijenborgh 7, 9747 AG Groningen, The Netherlands; 2 Department of Biological Sciences, Macquarie University, North Ryde, 2109 NSW Australia; 3 Groningen Institute for Evolutionary Life-Sciences, GELIFES, University of Groningen, Nijenborgh 7, 9747 AG Groningen, The Netherlands; 4 Royal Netherlands Institute for Sea Research, NIOZ, 1790 AB Den Burg, The Netherlands

**Keywords:** Infauna, Seagrass, Facilitation, Heatwave, Climate-change effects

## Abstract

Though there is mounting evidence that climate warming is altering trophic interactions between organisms, its effects on non-trophic interactions remain relatively undocumented. In seagrass systems, the bioturbating activity of infauna influences annual seagrass patch development by influencing seed burial depth and germination success as well as sediment properties. If bioturbation is altered by warming, consequences on seagrass may result. Here, we assessed how heatwaves alter seagrass seed burial depth and germination rates when no bioturbators (control), single bioturbators and mixtures of bioturbators of contrasting feeding activities are present. The three bioturbators manipulated were surface (top 1-2 cm of sediment) biodiffusor, the brown shrimp (*Crangon crangon*), the shallow (top 3–8 cm) diffusor, the common cockle, (*Cerastoderma edule*) and the upward (5–15 cm) conveyor, the polychaete, Cappitellidae spp. We applied two temperature treatments: (1) a present-day scenario set at the average summer temperature of seagrass habitat (17ºC); and (2) a heatwave scenario modelled on the maximum recorded temperature (26.6ºC). Under present-day conditions, seed burial was greater in the presence of bioturbators than the control where no infauna was added (42–74% *vs.* 33 ± 7%, respectively). Cockles had the greatest impact on seed burial amongst all the bioturbators. Under the heatwave scenario, seed burial in the mixed bioturbator treatment increased to match that of the cockle treatment. Cockles and polychaetes elevated the germination rates of buried seeds under present-day temperature, but not under the heatwave scenario. Overall, these results indicate that heatwaves have the potential both to amplify and disrupt non-trophic interactions, with implications for seagrass seed germination.

**Supplementary Information:**

The online version contains supplementary material available at 10.1007/s00442-025-05662-y.

## Introduction

One of the greatest current challenges for global change biology is to predict climate-change effects on biological interactions (Gilman et al. 2010; Boukal et al. 2019). Ecosystem structure and functioning is determined by ecological networks of interactions between organisms and their environment. Global warming not only changes the physical environment in which these interactions take place, but can modify these interactions by altering the distribution and abundance of species, their behavior, morphology and key physiological functions (Petchey et al. 1999; Gérard et al. 2020; Hamann et al. 2021) leading to cascading ecological effects over larger scales. For example, climate warming can create temporal, spatial and morphological mismatches between tightly co-evolved plants and pollinators (Gérard et al. 2020 and references therein), and it can alter trophic interactions, where predatory feeding rates increase but not the rates of prey reproduction (Brook 2009; Boukal et al. 2019).

Studies addressing the effects of climate warming on biotic interactions have, to date, focussed on trophic interactions, under the assumption that due to metabolic scaling with temperature (Brown et al. 2004; Ehnes et al. 2011) these will be particularly vulnerable to warming. Indeed, simplified modelling suggests that gradual warming will increase top-down control by trophic interactions, due to increased overall metabolic rates in consumer populations as well as increased variation in feeding rates at an individual scale (Gillooly et al. 2001; O’Connor 2009). Large effects of climate warming on non-trophic interactions may, however, result, where the altered environmental conditions disrupt facilitative and competitive interactions (Hoegh-Guldberg 1999) or modify ecosystem engineering. Ecosystem engineers (sensu Jones et al. 1994, 1997) are species that, by creating, destroying or modifying habitats, have large non-trophic effects on other organisms (Jones et al. 1994, 1997; Hastings et al. 2007; Olff et al. 2009). Climate warming may disrupt ecosystem engineering by causing collapse of populations of ecosystem engineers themselves (e.g. coral: Fordyce et al. 2019; Hoegh-Guldberg 1999; kelp: Filbee-Dexter et al. 2016; mussels: Nehls et al. 2006) or by modifying the environmental conditions that shape their interactions (McAfee et al. 2016). For example, where the role of ecosystem engineers derives in part from mitigation of environmental stressors, such as temperature, warming may enhance their positive species interactions (Bertness and Callaway 1994; Mulder et al. 2001; Lortie and Callaway 2006; Bulleri et al. 2018). There is a growing interest in how climate warming will affect non-trophic interactions, in particular the interactions between ecosystem engineers, in coastal ecosystems.

Ecosystem engineers, and their biological interactions, may be altered not only by gradual warming, but also by extreme temperature events (Wernberg et al. 2013; Pereira et al. 2015). Heatwaves have been increasing in frequency and intensity since the 1950s, leading to escalated environmental stress, especially for coastal systems (Jentsch et al. 2007; Murray and Ebi 2012). Sessile species in shallow coastal ecosystems are particularly sensitive to extreme temperatures, leaving significant long-term consequences that are hard to reverse. Seagrasses are ecosystem engineering marine foundation species that inhabit shallow coastal waters. They facilitate non-trophic interactions by creating a three-dimensional habitat for a range of marine fauna, attenuating hydrodynamic forces and protecting coastlines from erosion, and by promoting sequestration of particulate matter, including carbon. Amongst the species they facilitate are bioturbating, sediment-dwelling invertebrates (polychaetes, mollusks, and crustaceans) which, in reworking sediments, can determine sediment–water exchange as well as sediment properties such as grain size, organic content and oxygenation (Meysman et al. 2006; Solan and Herringshaw 2008; Shull et al. 2009).

The relationship between seagrass and invertebrates is, however, bi-directional. Bioturbation, along with other factors such as hydrodynamics, predation and turbidity (Moore et al. 1993; Brenchley and Probert 1998; Orth et al. 2000), can determine the germination and recruitment success of seagrasses. Bioturbators may directly affect seagrass germination success by influencing whether seeds are dislodged or embedded in a substrate and may indirectly influence germination by determining sediment conditions, including oxygenation and nutrient concentration (Dumbauld and Wyllie-Echeverria 2003; Cabaço et al. 2008; Valdemarsen et al. 2011; Johnson et al. 2018). Experimental studies suggest the greatest seagrass seed germination success can be obtained from seeds that are buried between 2 and 4 cm or shallower, into sediment column (Orth et al. 2000; Marion and Orth 2010; Jarvis and Moore 2015; Jørgensen et al. 2019). If seeds stay at the surface are buried too shallowly, they may be washed away and fail to germinate; if they are buried too deeply, they may experience adverse chemical conditions, or fail to reach the surface upon germination (Orth et al. 2000; Dumbauld and Wyllie-Echeverria 2003; Cabaço et al. 2008; Valdemarsen et al. 2011; Jarvis and Moore 2015; Statton et al. 2017). Seed germination may be strongly influenced by burial depth, even at the millimeter scale, due to the steep biogeochemical gradients that characterize sedimentary environments (Orth et al. 2000). Successful sexual reproduction resulting in high genetic diversity underpins seagrass’ resilience to global warming (Reusch 2006; Ehlers et al. 2008). Yet, based on the theory of increased metabolic activity at higher temperatures, the sediment reworking capacity, and thus the interaction between seagrass and bioturbating infauna may be highly susceptible to temperature (Fouw et al. 2016, El-Hacen et al. 2019). How warming will influence infauna-seagrass interactions is a topic requiring further research attention.

We experimentally tested how three common intertidal soft-sediment invertebrates of the Dutch Wadden Sea with contrasting feeding and, hence, bioturbation, traits independently and interactively influence *Zostera marina* seed burial and germination under ambient and heatwave scenarios. This benthic community is emmersed for several hours a day, making it susceptible to heatwaves, which have been increasing in frequency and duration in this area since 1901 (Heron et al. 2020). We hypothesized that bioturbation would increase under heatwave conditions leading to increased seed burial. However, we also hypothesized that whether this greater burial depth enhanced or diminished seagrass germination, would vary amongst bioturbator treatments, reflecting variation in the depths to which they move seeds.

## Materials and methods

### Experimental design 

Independent and interactive effects of bioturbators and temperature on *Zostera marina* seed burial and germination were tested in fully orthogonal lab experiments manipulating the presence of three common bioturbators of Dutch Wadden Sea tidal flats. Seed survival and germination are essential to annual intertidal *Z. marina* in the Wadden Sea. *Z. marina* seeds are easy to manipulate and observation time is relatively short (3 weeks to observe germination, according to Xu et al. 2016; Jørgensen et al. 2019).

The bioturbating taxa, selected based on their contrasting feeding and bioturbation traits, were: (1) the common cockle *Cerastoderma edule;* (2) the brown shrimp, *Crangon crangon,* and (3) marine polychaetes of Capitellidae family (dominated by *Heteromastus filiformis* and *Capitella* spp.). Brown shrimp are surface biodiffusers, organisms that disturb particles of the top layer in the sediment surface (0–1 cm depth). Common cockles are shallow biodifussers (0–5 cm), organisms that move particles in a random manner over short distances. Capitellid polychaetes belong to the conveyors, which are organisms that move particles from the surfaces of the sediment into the deeper layers, or vice versa, depending on the species (Aller and Yingst 1985, Chapin et al. 1992, Gerino et al. 2003). The dominant Capitellid species in this study convey sediment from 5 to 20 cm depth, towards the sediment surface (Linke 1939; Strömgren et al. 1973; Neira and Höpner 1994). Using these three taxa, five infaunal treatments were established: control (no animals), cockles only, shrimp only, polychaetes only, and all three infaunal taxa mixed (mixed infauna).

The five resulting infaunal treatments were crossed with two temperature treatments (ambient, heatwave) to give a total of ten treatments. The ambient temperature treatment was set at 17 ºC, the mean summer temperature in the Dutch Wadden Sea in 2017 (Rijkswaterstaat, Ministry of Infrastructure and Water Management). The heatwave simulation was modelled according to the maximum water temperature records in the area of 26.6ºC (Rijkswaterstaat, Ministry of Infrastructure and Water Management).

### Animals, sediment and seagrass collection

Sediments and invertebrates for use in laboratory experiments were collected at low tide in June 2020 from an intertidal flat near the barrier island of Schiermonnikoog, a national park in the Dutch Wadden Sea (53.47º N, 006.21º E). The collection site was approximately 250 m in length, and 80–90 m away from the saltmarsh fringing the tidal flat. Cockles were collected by hand, shrimp were collected from puddles of pooling water using a handheld shrimp net, while polychaetes were carefully excavated using a handheld sediment corer of 15-cm diameter which was pushed 20 cm into the sediment. Individuals of each species were generally juveniles, while adults were only occasionally encountered. Cockles and shrimp were transported back to the laboratory in coolers containing sea water that was continuously aerated. Sediment cores, for polychaete extraction, and also for supply of sediment to experimental units were transported on ice in coolers to the laboratory. In the laboratory, polychaetes were separated from sediments under a dissecting microscope.

Fauna were held in aerated tanks containing saline water (+ 30 ppt) while being exposed to light/dark cycle of 16/8 h and average ambient temperature of + 17ºC, until the start of the experiment. The sediments for the experimental units were prepared by: (1) removing the top 1 cm of surface sediments and wet sieving this through a 0.5-mm mesh to remove non-target macrofauna whilst retaining meiofaunal and microbial communities; and (2) autoclaving the remainder of sediments and flushing these twice with salt water (30 ppt) to lower the ammonium levels caused by decaying organic matter. In aquaria, autoclaved sediments formed the base of sediment cores, with the sifted and defaunated sediment forming the upper layer.

The *Zostera marina* seed-baring shoots (spathes) were collected in August 2019 from an intertidal meadow at Hamburger Hallig, in the German Wadden Sea (N54.35505°, E8.48467º). Spathes were transported from the field to laboratory in the Netherlands under refrigerated conditions (7ºC) within 24 h. In the laboratory, seeds were isolated by placing spathes in aerated seawater tanks and letting the negatively buoyant seeds sink. Seeds, separated from debris and other negatively buoyant organisms, were transferred to darkened tanks receiving a constant flow of aerated artificial seawater (30 ppt, Tropic Marin ©) and maintained at + 4 °C until the commencement of the experiment to prevent germination (Govers et al. 2022).

### Aquarium setup and sampling

The experiment was conducted in August and September 2020 in climate manipulation chambers in the aquarium at, Groningen Institute for Evolutionary Life Sciences, The Netherlands. Two replicate runs of the experiment were performed, each lasting 21 days under laboratory conditions allowing adequate time for seed germination (Xu et al. 2016; Jørgensen et al. 2019).

**Fig. 1:**
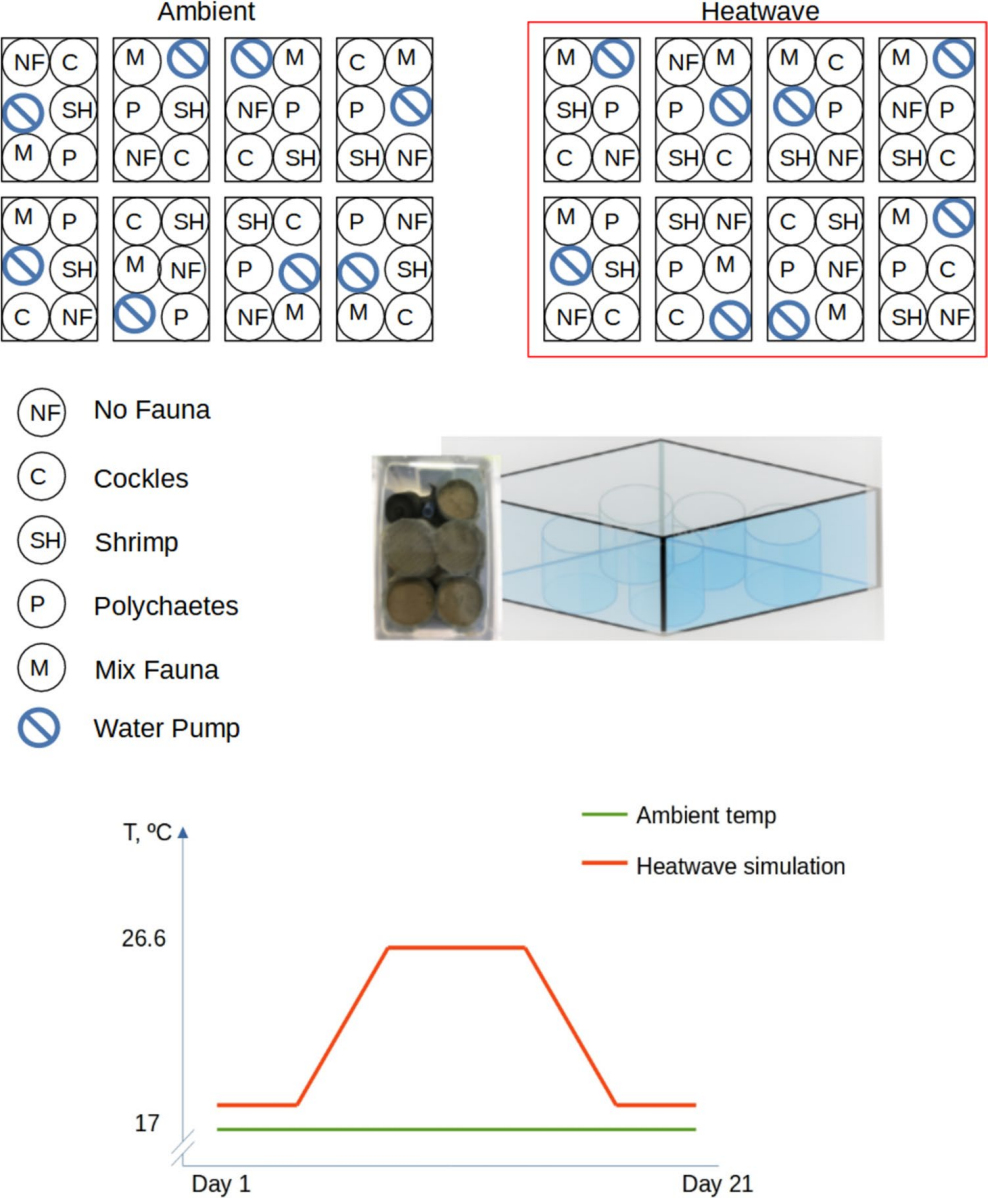
Schematic illustration of experimental set up of different fauna (*n* = 5) and temperature (*n* = 2, ambient and heatwave simulations) treatments through experimental run of 21 days

Within each experimental run, faunal effects on seed burial and germination were assessed in replicate 7.5-cm diameter open-ended polypropylene cylinders, filled with treated sediment cores of 6-cm depth and with 5 cm of overlying water. There were 80 replicate sediment cores per experimental run, divided amongst 16 15 × 26 × 17 cm tanks to give blocks of five cores. Within each of the tanks, one replicate core was randomly assigned to each of the five faunal treatments (cockles, shrimp, worms, mixed, control). Cores assigned to the cockle treatment received two cockles of 23.47 ± 0.19 mm (mean ± 1 SE) shell length, those assigned to the shrimp treatments received two juvenile shrimp of ~ 20-mm length, while those assigned to the polychaete treatment received 5–7 individuals of 10–20 mm length, matching natural densities in the Dutch Wadden Sea. The mixed fauna treatment comprised 1 cockle, 1 shrimp and 3–4 polychaetes, where the proportion of each organism were adjusted to match the total organism biovolume. The control treatment contained no added fauna (Fig 1).

**Fig. 2:**
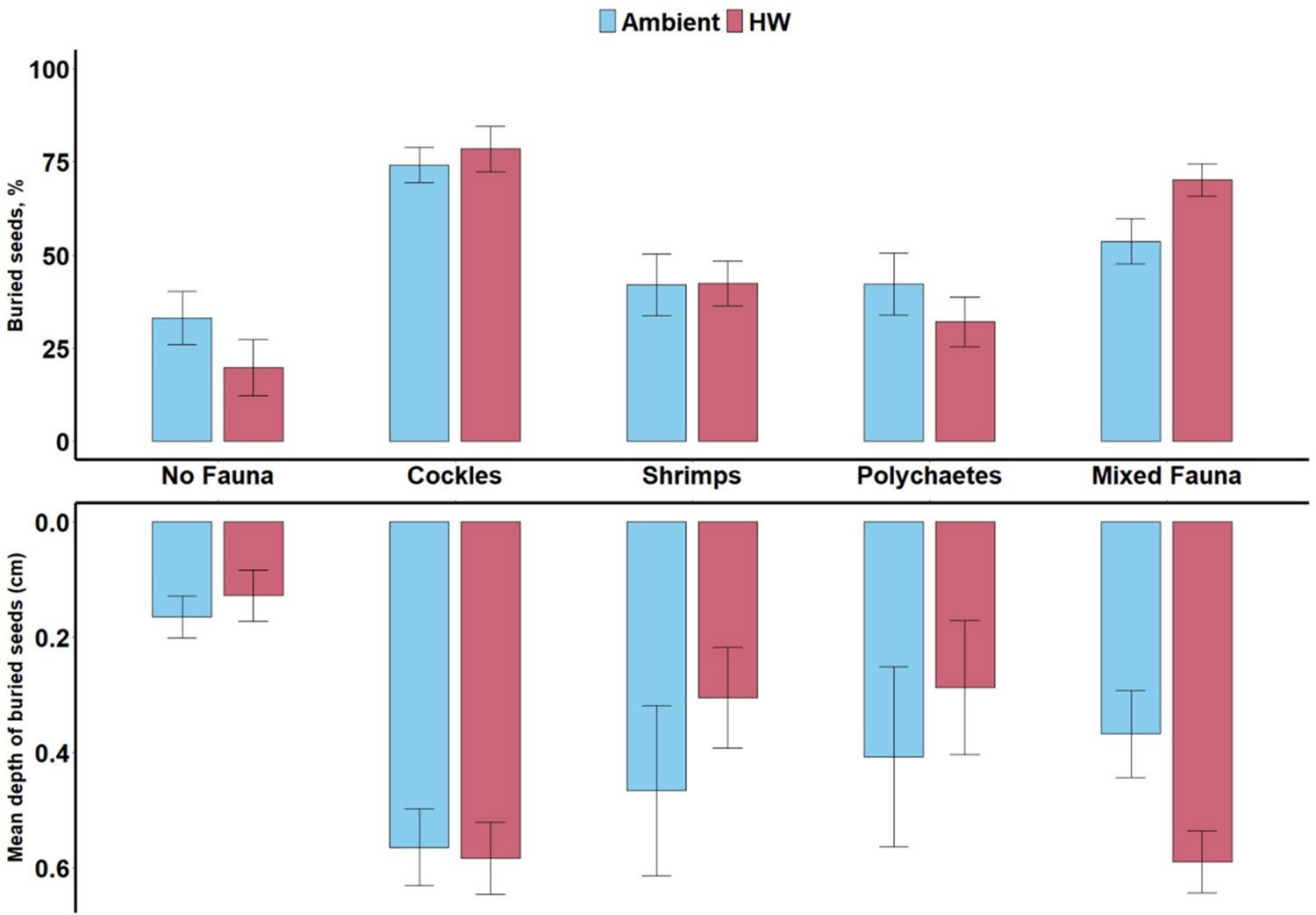
Effects of fauna (*n* = 5) and temperature (*n* = 2, ambient, and heatwave, HW) on *Z. marina* seed burial, as measured by the percentage (%) of total seeds counted that were found below the surface (above) and weighted mean depths (below); bars are means ± SE

Species in their juvenile life stage were chosen for the experiment due to their abundance at the collection site, adults were only occasionally encountered during sampling. Eight replicate tanks were randomly assigned to the ambient temperature and 8 to the heatwave simulation(Jørgensen et al. 2019) and placed in two larger shallow tanks half-filled with tap water that was either maintained at the ambient temperature of 17ºC (ambient treatment) or heated using an electric water heater with an integrated manual thermostat (heat-wave treatment). The external water temperature control set up ensured the simultaneous water temperature alterations in each experimental treatment tank. The water surrounding the ambient temperature treatments was kept at a steady temperature of 17ºC throughout the experiment. For the heat-wave treatment, ambient water conditions of 17ºC were maintained for the first 6 days, before temperature was slowly ramped up, at 2ºC per day, for 5 days, to reach a stable maximum temperature of 26.6 ºC which was maintained for 6 days, before 5 days of decreasing the temperature at a rate of 2 ºC per day, and 4 days again at the ambient temperature of 17 ºC (Fig. 2). Throughout the experiment, a light/dark cycle of 16/8 h was maintained, to represent the average summer daylight duration in the Netherlands.

Tanks with treatments received artificial seawater which was maintained at a salinity of 30 ppt, and nitrate and ammonia levels of 0.344 ± 0.077 mg/l and 0.030 ± 0.038 mg/l, respectively, approximating the chemistry of Wadden Sea water samples (mg NO_3_/l: 0.238 ± 0.066, mg NH_4_/l: 0.176 ± 0.004). Nitrate and ammonia levels in experimental units were checked daily using sensitive quick water quality measure kits (Sera GmbH) with water exchanged using slow speed aquarium pumps every 2 to 3 days to prevent build-up of waste products. The temperature and salinity of four randomly selected tanks per temperature treatment were determined daily using a handheld universal multi-parameter sensor (WTW ProfiLine pH/Cond 3320 Multi-Parameter Portable Meter, Xylem), confirming that desired experimental conditions were met.

Infauna were acclimated to experimental conditions in cylinders (initially 17 °C for both temperature treatments) for 48 h prior to the addition of seagrass seeds. At the end of the acclimation period, ten *Z. marina* seeds (3.48 ± 0.06 mm in length) that were dark in color and firm-to-touch (i.e. alive but ungerminated), were added to each replicate core by spreading the seeds on the sediment surface. During the 21 day experiment, the invertebrates were fed every 2 days with life phytoplankton (cultivated nannochloropsis, Colombo Live Phytoplankton), by mixing 2 ml of feed in 20 ml of sea water and pouring 1 ml of the solution into each tank.

At the conclusion of the 21 day experiment, the position of seeds in the sediment, and the status of each (germinated, ungerminated but alive, ungerminated but dead) was assessed visually, with the aid of a dissecting microscope, and by touch (firm-to touch or hollow). Germinated seeds had a cracked seed coat with emerging radicle (primary root), seeds that were firm to touch were assumed to be ungerminated but alive (viable), while ungerminated but dead seeds had empty seed coats which broke when depressed. First, unburied *Z. marina* on surface of the sediment were collected and enumerated by status. Second, each sediment core was divided into six 1-cm deep strata, each of which was separately sieved over a 0.4-mm mesh. The material retained on the mesh was searched for live invertebrates, which were identified and counted, and seeds which were enumerated according to germination status.

### Data analysis

For each replicate core, the following four metrics were calculated using seed recoveries from the various depth strata: (1) the total number of seeds recovered from the surface of the sediment; (2) the total number of seeds recovered from below the sediment surface; (3) the weighted average mean depth (calculated using mean strata depths) seeds were buried; (4) the percentage of surface seeds that were germinated; and (5) the percentage of buried seeds that were germinated.

Generalized linear models assessed effects of experimental treatments (fauna and temperature) on the percentage of seeds buried and the weighted mean depth (cm) of their burial, while linear mixed effects models uncovered differences amongst the treatments in seed germination success (%), accounting for variation amongst individual samples. These analyses had four factors: infauna (five levels: no fauna, cockles, shrimp, polychaetes, mixed fauna); temperature (two levels: ambient, heatwave), block (random; 8 levels for each temperature treatment), and experimental run (random, 2 levels). Whereas the statistical assumptions of data normality and homogeneity of variances were not met, the data were transformed using square root (seed burial depth data), as well as arc-sine transformations (asin(response value)^0.25, seed germination data). Significant effects of treatment differences were evaluated using model summaries (function summary(x)). All data analysis was conducted in R Studio (Version 1.4.1717), using packages Car (version 3.0–10, Fox et al., 2020) and lme4, ggplot2 (version 3.3.3, Wickham et al. 2020) for graphical illustrations of the results, and the core stats package (version 4.0.3, R core team 2020).

## Results

All invertebrates survived each of the experimental runs. Seed recovery for run 1 was 96.2% (No Fauna: 91%, Cockles: 100%, Shrimp: 96%, Polychaetes: 95%, Mix Fauna: 99%), and for run 2 was 92.8% (No Fauna: 86%, Cockles: 95%, Shrimp: 99%, Polychaetes: 87%, Mix Fauna: 97%). Percent seagrass seed germination was greater and mortality lower for run 1 (germination: 15%, mortality: 19.23%) than run 2 (germination: 7.2%, mortality: 24.43%).

### Seed burial

Few seeds were found in the deepest sediment layers, with most (91%) found within the top centimeter of sediment (Fig. 2). The number of seeds buried and the mean depth to which they buried was determined by the interacting effects of faunal and temperature treatments (Table 1; Fig. 2). Only few seeds were buried onto the deepest sediment core layers, whereas the majority of seeds were contained just below the surface, within the first centimeter of sediment core (Fig. 2).

**Table 1:** Summary of Tukey post-hoc tests examining sources of significant treatment effects of fauna and temperature on percentage (%) of buried seeds, their mean burial depths (cm), and percentage (%) of germinated seeds across faunal treatments and temperatures. Contrasts within treatments are as compared to the fauna-free control

Control	Temperature	Cockles	Shrimp	Polychaetes	Mix fauna
Buried seeds (%)	Ambient	t = 4.0, p < 0.001	t = 1.4, *p* = 0.162	t = 1.5, *p* = 0.126	t = 2.6, *p* = 0.011
	Heatwave	t = 1.7, *p* = 0.098	t = 1.3, *p* = 0.195	t = 0.5, p = 0.645	t = 2.2, *p* = 0.032
Seed burial depths	Ambient	t = 4.2, *p* < 0.001	t = 2.3, *p* = 0.021	t = 1.8, *p* = 0.067	t = 2.4, *p* = 0.017
	Heatwave	t = 0.8, *p* = 0.409	t = 0.3, *p* = 0.787	t = −0.1, *p* = 0.938	t = 2.2, *p* = 0.032
Buried seed germination success (%)	Ambient	t = 2.4, *p* = 0.017	t = 0.8, *p* = 0.417	t = 2.4, *p* = 0.017	t = 1.8, *p* = 0.073
	Heatwave	t = 1.6, *p* = 0.114	t = 0.5, *p* = 0.622	t = 2.1, *p* = 0.040	t = 1.7, *p* = 0.091

Under ambient conditions, a greater percentage of seeds were buried in cockle (74 ± 5%) and mixed species (54 ± 6%) than control treatments (33 ± 7%; Table 1, Fig. 2, Table S1) and seeds were buried to a greater depth, in treatments with than without fauna (Table 1, Fig. 2). Under heatwave conditions, there was similarly a greater percentage of seeds buried in the mixed species (70 ± 4%) than the control treatment (20 ± 7.5%; Table 1, Fig. 2, Table S1), with their burial depth also significantly greater in the mixed species than control treatment (Table 1, Fig. 2). Overall, amongst faunal treatments, the number of seeds that were buried and the depth to which they were buried was greatest in the cockle treatment (Fig. 2, Table S1), with seed burial in the cockle treatment unaffected by warming (Fig. 2). Only in the mixed fauna treatment did seed burial display an effect of warming, with warming increasing the number of seeds buried from 54 ± 6% to 70 ± 4%, and their mean burial depth from 0.37 ± 0.08 cm to 0.59 ± 0.05 cm to match that of the cockle-only treatment (Fig. 2, Table S1).

**Fig. 3:**
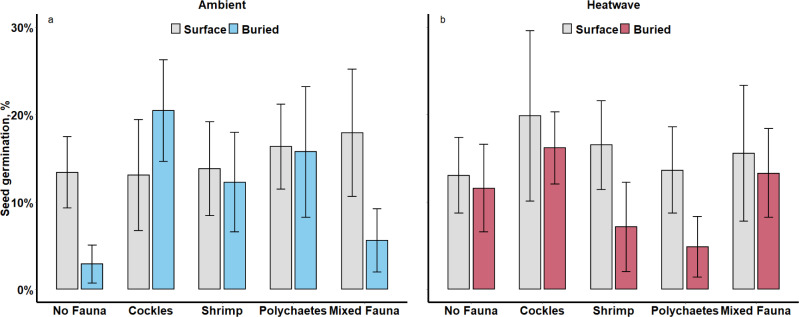
Percentage (%) of unburied (surface) and buried (sub-surface) *Z. marina* seeds that successfully germinated, when exposed to varying faunal treatments (*n* = 5), and when exposed to ambient temperature (**a**) or to a heatwave (**b**); bars are means ± SE

Effects of fauna on seagrass germination varied between ambient and heatwave scenarios (Fig. 3). Under ambient conditions, germination rates in replicates without fauna were lower for the buried than the unburied seeds (marginal trend: t = 1.8, p = 0.070, Table S2); while in replicates with cockles germination rates displayed the reverse pattern, being greater when seeds were buried (seed burial: cockle addition: t = 2.4, p = 0.017; Fig. 3a, Table 1, Table S2). Adding other fauna did not influence the effect of seed burial on germination rates (compared to seeds at No Fauna treatment on sediment surface, seed burial: shrimp addition: t = 1.2, *p* = 0.237; seed burial: polychaetes addition: *t* = 1.2, *p* = 0.238; seed burial: mixed fauna: t = 0.1, *p* = 0.882, Table S2). However, the germination rates of the buried seeds were higher when cockles or polychaetes were added to the sediment compared to the cores without any added fauna (no added fauna vs. cockles: t = 2.1, *p* = 0.040; and polychaetes: t = 2,4, *p* = 0.017; Table 1, Fig. 3a, Table S3). The heatwave eliminated all the positive effects of bioturbating fauna on germination rates. Instead, adding polychaetes to the sediment contributed to a significant decrease in germination success of the buried seeds (polychaetes addition: heatwave: t = 2.1, p = 0.040; Fig. 3b, Table 1, Table S3). There were no effects of adding fauna on the germination rates of the seeds remaining on the surface of the cores for either of the temperature treatments (Table S2).

## Discussion

Identifying how ecological relationships change with temperature is a crucial step towards understanding the consequences of climate change on ecosystem functioning. Where ecosystem engineering is influenced by warming, large impacts on biological non-trophic interactions may result (Byers et al. 2006; Zhou et al. 2022). On the land and in the sea, seed burial influences both small- and large-scale distributions and abundances of adult plants (Warr et al. 1993). Alongside abiotic processes and seed-mediated burial, the activities of fauna are critical to seed burial processes (Chambers and MacMahon 1994; Blackburn and Orth 2013; Zhu et al. 2016a). In marine sediments, bioturbating infauna can dislodge seeds or, alternatively bury seeds below their critical depths (Philippart 1994; Valdemarsen et al. 2011; Blackburn and Orth 2013), and as a metabolically driven process, their activity may be expected to change as the climate warms. In this study we found strong interacting effects of bioturbators and heatwaves on seagrass seed burial and germination. These results suggest that where germination rate is limiting of adult seagrass populations, climate warming may have large indirect effects on seagrass.

How infaunal invertebrates ecologically engineer their sediment environments is dependent on functional traits of infaunal species, such as their feeding guild, mode of locomotion and morphology (Aller 1982; Gerino et al. 2003; Jumars et al. 2015). Though all three infaunal species examined by this study enhanced seed burial over what was observed in control (fauna-free) sediment, as in previous studies (Blackburn and Orth 2013; Zhu et al. 2016a; Li et al. 2017) the magnitude of this effect varied with functional group. Despite predictions, based on previous studies (e.g. (Mermillod-Blondin et al. 2004; Delefosse and Kristensen 2012; Blackburn and Orth 2013; Zhu et al. 2016b, a; Schenone et al. 2019) that polychaetes might have particularly strong effects on seed burial, to the contrary it was cockles that had the greatest effect. This may reflect the larger body size of the cockles as compared to the other bioturbators manipulated in this study, as biomass is generally a good predictor of sediment reworking potential (Braeckman et al. 2010; Valdemarsen et al. 2011). In addition, it may reflect functional differences between the taxa. Though upward conveying polychaetes oxygenate deeper sediment layers while ventilating their burrows (Michaud et al. 2005; Schenone et al. 2019), their sediment particle displacement capacity is limited mostly to the formation of the burrows (Kristensen et al. 2012). This may result in smaller volume of sediment displacement and reduces the likelihood of affecting seeds. On a contrary, cockles as biodifussers, have lower capacity to oxygenate deeper sediments but are more efficient in mixing sediment particles up to their burial depth (~ 2 cm, depending on cockle size, Cozzoli et al. 2020; Li et al. 2017; Mermillod-Blondin et al. 2004). We observed cockle burying and reburying in most replicate cores.

Despite predictions that heatwaves might universally affect infaunal activity, and hence seed burial, we only found an effect of the heatwave on seed burial in the mixed species treatment. Ecological interactions such as competition, facilitation and predation may dampen or exacerbate stressor impacts (Christensen et al. 2006; Blake and Duffy 2010; Hicks et al. 2011). In addition, biomass compensation between single and mixed species treatments may lead to differences in densities of individual taxa that may also contribute (Jolliffe 2000; Facelli 2003). In our experimental treatments, cockles exhibited the highest seed burial potential, but this potential decreased in the presence of other fauna or when cockle density was reduced (e.g., 1 cockle in polyculture vs. 2 in monoculture). Whilst this suggests density effects could be a contributing factor, additional mechanisms such as altered behaviour under species mixing or stress from heatwaves may also play a role. Direct assessments of changes to the behaviors of macrofauna in heatwaves would assist in determining the natural mechanism for this species mixing effect. Irrespective of the mechanism, this result supports a growing literature demonstrating differential effects of environmental stressors on species when they are within *vs.* isolated from their ecological communities (Petchey 2004; Nagelkerken and Munday 2016; Carrier-Belleau et al. 2021). Our results highlight the importance of testing ecological responses to environmental change using communities rather than individual organisms, as buffering or multiplicative effects may be seen (Duggins 1981; Gilman et al. 2010; Kordas et al. 2011; Wernberg et al. 2012). To further understand the observed differences between monocultures and polycultures, future work should focus on investigating the specific mechanisms driving these effects, including both intraspecific and interspecific interactions, as well as changes in behaviour under varying environmental conditions.

Seed burial may influence seed survival, and hence also germination, by influencing seed anchoring and retention at suitable settlement sites, by providing escape from predation, and by placing seeds in suitable sediment conditions (Chambers and MacMahon 1994; Zhu et al. 2016a; Jørgensen et al. 2019). In our laboratory experiment, in which seeds were free of predators and strong hydrodynamic forces, seeds on the sediment surface overall had a higher rate of germination than buried seeds. This pattern of greater germination at the surface was also found for *Zostera nigricaulis* and *Zostera marina* seagrass seeds in similar mesocosm experiments (Cumming et al. 2017; Jørgensen et al. 2019). Other studies with *Z. marina*, however, found that seeds placed at or near the sediment surface had delayed germination, with optimal germination success achieved for shallowly buried seeds (Moore et al. 1993; Jarvis and Moore 2015). The variable effects of seed burial on germination may indicate population (Jarvis and Moore 2015) and species-level (Orth et al. 2000) variation in the optimal conditions for germination and/or variation amongst studies in sediment conditions, that determine their suitability for germination. Despite the controlled experimental conditions of our study, we found up to 13% of seeds were lost during an experimental runs in defaunated treatments, up to 11% in treatments with polychaetes, and 1–3% in the rest of the faunal treatments, probably through the process of water renewal. This suggests that in natural systems, with greater hydrodynamic forces, transport of unburied seeds may be considerable, highlighting the importance of burial to the seed bank.

One of the most widely investigated roles of bioturbators in soft bottom systems is the improvement of sediment quality, by facilitating nutrient and gas exchange between sediment layers and overlying water column, as well as sediment enrichment with nutrients via biodeposition (Reusch et al. 1994; Braeckman et al. 2010; Mermillod-Blondin 2011). Although we did not measure changes in sediment conditions in response to bioturbators, our results support a role for bioturbators influencing sediment suitability for germination. Under ambient conditions, the lack of positive effect of seed burial on germination seen in the absence of fauna was not replicated when infauna were present. To the contrary, in treatments with infauna we observed positive effects of burial on seed germination. Under the heat-wave scenario, though, such positive effects of seed burial disappeared, and to the contrary, seed germination was negatively affected by burial in the polychaete treatment. High lugworm (*Arenicola marina*) densities have been implicated in the decline of *Zostera marina* in the Dutch Wadden Sea (Valdemarsen et al. 2011; Govers et al. 2014). The results suggest that the effects of bioturbators on seagrass habitats might be of a fine balance between improving the sediment suitability for plant growth and hampering seagrass establishment by removing the seeds from their optimal germination conditions.

Overall this study highlights the potential for strong ecosystem level effects of heat-waves on seagrass to arise from changes in non-trophic interactions. We demonstrated that seed burial and germination is influenced by the bioturbating activities of sediment-living invertebrates and these processes are altered by realistic warming events. Yet, the extent of heat impact on this interaction depended on the functional composition of the bioturbating community, as the increased capacity to bury seagrass propagules due to warming was only observed in the diverse fauna treatment where we mimicked a more natural composition of bioturbation traits by mingling species of different functional groups. In natural ecosystems, bioturbators of distinct functional traits coexist in wide varieties. Moreover, effects of seed burial on germination, and seedling development, may be dependent on local biological and physical factors, such as the abundance and foraging method of seed predators, the magnitude of waves and currents that can dislodge seeds, as well as sediment properties. Field and laboratory tests are required across a boarder range of conditions to understand thresholds beyond which major shifts in seagrass ecosystems will occur.

There is growing evidence from terrestrial (Berg et al. 2010), freshwater (Beebee 1995) and marine environments (Draper and Weissburg 2019), that global warming can have large disruptive effects on ecosystems arising from the modification of species interactions (Memmott et al. 2007; Filbee-Dexter et al. 2016). Though most studies have focussed on disruptions to trophic interactions (Ings et al. 2009; Ellison 2019), our study highlights the need to consider non-tropic interactions too. Small-scale experiments are useful in developing mechanistic understanding of non-trophic interactions and their sensitivity to environmental change. However, these need to be coupled with field observations of shifts in ecosystems following major weather events to understand the land- and sea-scape ramifications of shifting ecological interactions. The understanding thereby developed can guide conservation, restoration, and eco-engineering efforts aimed at building ecosystem resilience.

## Supplementary Information

Below is the link to the electronic supplementary material.

Supplementary file1 (PDF 82 KB)

## Data Availability

The data was deposited in GitHub.com under the references https://github.com/SimonaLaukaityte/heatwave-on-animal-plant-interactions/blob/ea4679016e8bddcd083cd93fe2fd6f33b6b1025e/data.txt and https://github.com/SimonaLaukaityte/heatwave-on-animal-plant-interactions/blob/ea4679016e8bddcd083cd93fe2fd6f33b6b1025e/depth_data.txt

## Code availability

Code was deposited in GitHub.com under the reference https://github.com/SimonaLaukaityte/heatwave-on-animal-plant-interactions/blob/14286c76bf40a5416060b32e29c9e57f9ec51bd2/Analyses_Klemens_Simona_2022_01_28.R
