# Supplementary material for: Warming alters non-trophic interactions in soft bottom habitats
Source: Oecologia. 2025 Feb 6;207(2):30. doi: 10.1007/s00442-025-05662-y (PMC11799095; doi:10.1007/s00442-025-05662-y)
Supplement: Supplementary file 1 — Supplementary file1 (PDF 82 KB) [file 442_2025_5662_MOESM1_ESM.pdf]

Title: Warming alters non-trophic interactions in soft bottom habitats.

Authors: Simona Laukaityte, Melanie J. Bishop, Laura L. Govers, Britas D.H. Klemens Eriksson.

Corresponding author: S. Laukaityte@rug.nl

All authors declare that they have no conflicts of interest.

Table S1: percentage (%) of buried seeds, germinated seeds and mean seed burial depths ± SE (cm) across faunal treatments and temperatures (ambient: 17°C, heatwave 26.6°C)

**Fauna treatment**	**Temperature treatment**	**Seed burial, %**	**Germination success, %**	**Mean seed depth, cm**
**No fauna**	Ambient	33.07 ± 7.21	2.34 ± 1.79	0.17 ± 0.04
	Heatwave	19.73 ± 7.52	5.59 ± 2.85	0.13 ± 0.04
**Cockles**	Ambient	74.19 ± 4.68	17.27 ± 4.94	0.57 ± 0.07
	Heatwave	78.50 ± 6.05	14.11 ± 3.58	0.58 ± 0.06
**Shrimp**	Ambient	41.99 ± 8.39	10.11 ± 5.02	0.47 ± 0.15
	Heatwave	42.41 ± 5.94	5.21 ± 3.83	0.31 ± 0.09
**Polychaetes**	Ambient	43.61 ± 8.73	10.54 ± 4.04	0.41 ± 0.16
	Heatwave	31.36 ± 6.34	2.94 ± 2.12	0.29 ± 0.12
**Mix fauna**	Ambient	53.68 ± 6.08	2.92 ± 1.70	0.37 ± 0.08
	Heatwave	70.17 ± 4.41	10.66 ± 4.67	0.59 ± 0.05

All authors declare that they have no conflicts of interest.

Table S2: Model output of seed germination success (%), compared against germinated seeds in fauna-free treatment (No Fauna) at sediment surface in ambient temperature (intercept). Model: asin(value1^0.25) ~ Germination % * fauna * temp + run + (temp/block). Significant values that were used in the text are in bold.

	Value	Std.Error	DF	t-value	p-value
(Intercept)	0.92	0.167	147	5.49	<0.001
**Germination_buried seeds**	**-0.29**	**0.159**	**114**	**-1.83**	**0.070**
**Cockle**	**-0.13**	**0.159**	**147**	**-0.82**	**0.415**
**Mix**	**0.01**	**0.151**	**147**	**0.04**	**0.966**
**Shrimp**	**-0.06**	**0.156**	**147**	**-0.40**	**0.690**
**Polychaete**	**0.04**	**0.153**	**147**	**0.28**	**0.777**
**Warm**	**-0.04**	**0.168**	**147**	**-0.26**	**0.792**
**run**	**-0.21**	**0.053**	**147**	**-4.01**	**<0.001**
**Germination_buried seeds:Cockle**	**0.54**	**0.223**	**114**	**2.42**	**0.017**
Germination_buried seeds:Mix	0.03	0.218	114	0.15	0.882
Germination_buried seeds:Shrimp	0.27	0.225	114	1.19	0.237
Germination_buried seeds:Polychaete	0.26	0.223	114	1.18	0.239
Germination_buried seeds:WARM	0.31	0.244	114	1.27	0.205
Cockle:tempWARM	0.20	0.231	147	0.86	0.390
Mix:tempWARM	-0.04	0.215	147	-0.16	0.871
Shrimp:tempWARM	0.13	0.219	147	0.59	0.559
Polychaete:tempWARM	-0.05	0.215	147	-0.23	0.820
COLD:block	0.01	0.008	147	1.30	0.196
tempWARM:block	<0.01	0.006	147	0.49	0.626
Germination_buried seeds:Cockle:tempWARM	-0.51	0.334	114	-1.52	0.130
Germination_buried seeds:Mix:tempWARM	-0.03	0.323	114	-0.10	0.921
Germination_buried seeds:Shrimp:tempWARM	-0.58	0.330	114	-1.75	0.083
Germination_buried seeds:Polychaete:tempWARM	-0.51	0.331	114	-1.53	0.129

All authors declare that they have no conflicts of interest.

Table S3: model output of seed germination success (%), compared against buried seeds that germinated in fauna-free treatment (No Fauna) at ambient temperature (intercept). Model: asin(value1^0.25) ~ Germination% * fauna * temp * run + (temp/block). Significant values that were used in the text are in bold.

	Value	Std.Error	DF	t-value	p-value
(Intercept)	0.31	0.610	138	0.51	0.613
Germination_unburied seeds	1.60	0.819	104	1.95	0.054
Cockle	1.42	0.811	138	1.75	0.082
Mix	0.69	0.811	138	0.85	0.395
Shrimp	0.06	0.851	138	0.07	0.944
Polychaete	1.33	0.836	138	1.60	0.113
tempWARM	1.05	0.986	138	1.07	0.288
run	-0.09	0.237	138	-0.39	0.698
Germination_unburied seeds:Cockle	**-2.38**	**1.143**	**104**	**-2.08**	**0.040**
Germination_unburied seeds:Mix	-2.02	1.117	104	-1.81	0.073
Germination_unburied seeds:Shrimp	-0.94	1.160	104	-0.81	0.417
**Germination_unburied seeds:Polychaete**	**-2.78**	**1.148**	**104**	**-2.42**	**0.017**
Germination_unburied seeds:tempWARM	-2.58	1.246	104	-2.07	0.041
Cockle:tempWARM	-1.96	1.235	138	-1.59	0.114
Mix:tempWARM	-2.10	1.235	138	-1.70	0.091
Shrimp:tempWARM	-0.63	1.278	138	-0.49	0.622
**Polychaete:tempWARM**	**-2.67**	**1.289**	**138**	**-2.07**	**0.040**
Germination_unburied seeds:run	-0.53	0.320	104	-1.64	0.104
Cockle:run	-0.40	0.315	138	-1.28	0.204
Mix:run	-0.26	0.315	138	-0.82	0.413
Shrimp:run	0.05	0.327	138	0.17	0.867
Polychate:run	-0.41	0.325	138	-1.26	0.211
tempWARM:run	-0.31	0.383	138	-0.81	0.417
tempCOLD:block	0.01	0.009	138	1.61	0.110
tempWARM:block	<0.01	0.006	138	0.63	0.532
Germination_unburied seeds: Cockle:tempWARM	4.19	1.699	104	2.46	0.015
Germination_unburied seeds: Mix:tempWARM	3.58	1.654	104	2.16	0.033
Germination_unburied seeds: Shrimp:tempWARM	1.87	1.691	104	1.10	0.272
Germination_unburied seeds:	4.89	1.695	104	2.89	0.005

All authors declare that they have no conflicts of interest.

Polychate:tempWARM					
Germination_unburied seeds: Cockle:run	0.74	0.450	104	1.64	0.1045
Germination_unburied seeds:Mix:run	0.80	0.438	104	1.83	0.070
Germination_unburied seeds:Shrimp:run	0.28	0.454	104	0.61	0.544
Germination_unburied seeds:Polychate:run	1.01	0.449	104	2.25	0.027
Germination_unburied seeds:tempWARM:run	0.91	0.490	104	1.86	0.066
Cockle:tempWARM:run	0.66	0.483	138	1.36	0.176
Mix:tempWARM:run	0.81	0.483	138	1.68	0.096
Shrimp:tempWARM:run	0.07	0.497	138	0.15	0.881
Polychate:tempWARM:run	0.84	0.504	138	1.68	0.096
Germination_unburied seeds: Cockle:tempWARM:run	-1.48	0.671	104	-2.21	0.030
Germination_unburied seeds:Mix:tempWARM:run	-1.42	0.649	104	-2.20	0.030
Germination_unburied seeds:Shrimp:tempWARM:run	-0.52	0.665	104	-0.79	0.434
Germination_unburied seeds:Polychate:tempWARM:run	-1.76	0.665	104	-2.64	0.009

All authors declare that they have no conflicts of interest.
